# A Detailed Description and Discussion on Conjoined Twins

**DOI:** 10.7759/cureus.29526

**Published:** 2022-09-24

**Authors:** Aabhas Bindlish, Anupama Sawal

**Affiliations:** 1 Anatomy, Jawaharlal Nehru Medical College, Datta Meghe Institute of Medical Sciences, Wardha, IND

**Keywords:** embryological abnormalities, sacrifice surgery, craniopagus twins, thoracopagus twins, siamese twins, conjoined twins

## Abstract

Conjoined twins are described as having been physically fused during pregnancy and delivery. They were first mentioned a long time ago when there was not much known about this. They share some organs that are vital for survival, like the heart; these twins are almost impossible to save, but there are some cases wherein there is evidence of their survival. The article aims to present a unique discussion on conjoined twins. This article talks about the formation of conjoint twins, their types and nomenclatures, embryological concepts, past history/traditional tales, case studies, and the medical enhancements happening in this area. Both fission and fusion are thought to contribute to the disease. A monozygotic twin pregnancy cleaves when it occurs more than thirteen days after fertilization. There is just one placenta and one womb for conjoined twins (one amniotic sac). The twins that are born and stay alive after delivery usually stay alive for a few days or weeks. It’s pretty rare for them to live a long prosperous life, but this article shows the otherwise, too, like the Siamese twins, which is a unique example of conjoint twins who lived for a long time. This kind of pregnancy is a complex procedure that needs to be managed by a team of professionals.

## Introduction and background

Due to the imperfect division of one fertilised ovum, conjoined twins are identical monozygotic twins that do not entirely separate from one another but are still partially linked to one another. Conjoined twins are monochorionic (one placenta) and monoamniotic (one amniotic sac). This condition is proposed to have resulted from either fission or fusion [1]. On the other hand, the fission and fusion theories do not account for every possible conjunction and cannot be applied to the full range of findings in conjoined twins [2].

Since the dawn of time, conjoined twins have fascinated both the general public and the medical community. Their arrival was initially perceived as a warning of approaching doom. Then there was a protracted time during the Middle Ages and into the nineteenth century when they were viewed as oddities or monstrosities and were displayed at circuses and sideshows for a significant financial reward [3]. Conjoined twins have received a lot of media attention recently, which has coincided with an increase in their success in being separated due to advancements in medical technology [4].

Conjoint twins are also popularly known as Siamese twins as they were one of the longest-living conjoined twins at 63 years of age. The celebrated conjoined Chinese twins Chang and Eng were born in Siam (Thailand) and got famous while working in an international circus. They were thoracopagus (explained later in the article) and shared a common liver [5].

## Review

Case study

A famous case published in the British medical journal showed a thoracopagus wherein a shared umbilicus was externally connected to each side's manubrium sterni. The external organs exhibited a complexity that would have made surgical separation near impossible. A pharynx, larynx, and upper trachea were provided to each side of the thoracopagus. At the maximum point of external conjunction, the trachea on one side and the oesophagus on the other are connected to form a single tube. Once within a single stomach, which ultimately emptied into a single jejunum, this tube travelled via a connected diaphragm. However, the ileum then split again at the location of a single Meckel's diverticulum to pass separately on each side into a normal caecum. In order to prevent the surgical liver division from cutting across biliary and venous pathways on both sides, the liver was widely connected, with the plane of conjunction running obliquely. One of the twins had a single heart in the thorax from which two distinct aortas emerged asymmetrically to give circulation, one aorta for each side. The configuration of the prominent veins and the heart chambers was exceedingly intricate and had not yet undergone complete analysis. A peculiar finding was the emergence in the thoracic cavity of several distinct and isolated patches of collapsed lung tissue that may have been mistaken for pulmonary bronchial segments. This is puzzling, given that lung alveoli sprout out from developing bronchi [6].

Traditional tales

**Figure 1:**
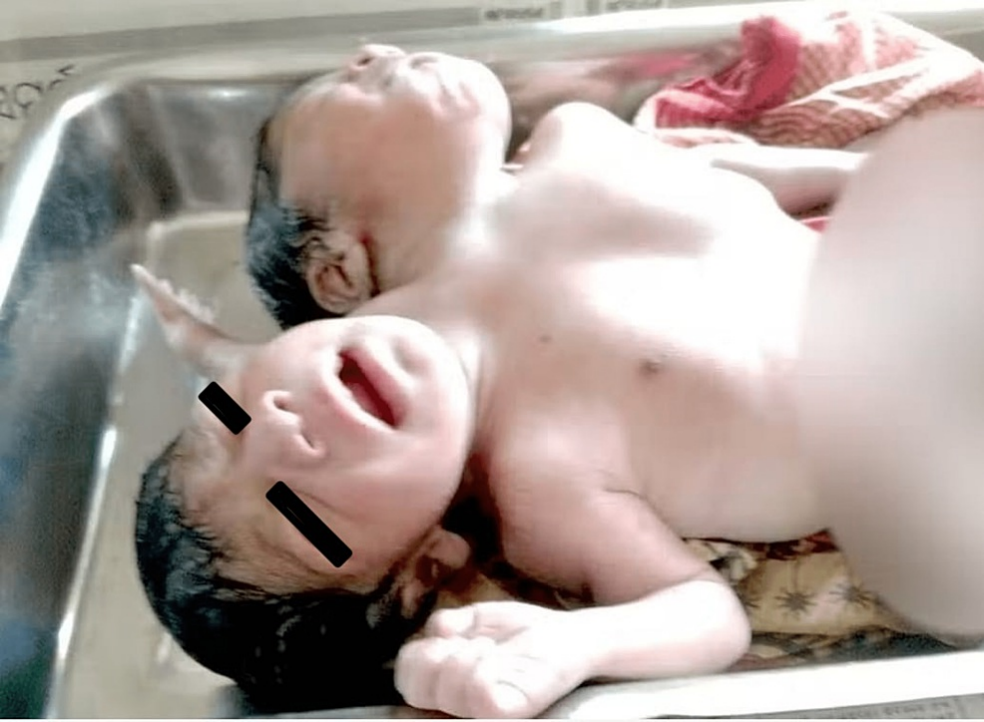
Siamese twins Conjoined twins with two heads, three arms, and a torso Image credit: Author Aabhas Bindlish

According to tradition, Mary and Eliza Chulkhurst, the Biddenden Maids, were born in the year 1100 to relatively wealthy parents [7]. Their shoulders and hips were connected together. Even though they occasionally differed on little topics and frequently quarrelled, which sometimes resulted in blows, they were naturally excellent friends. When the Maids had been living together for 34 years in 1134, Mary became ill out of the blue and passed away. Eliza was offered a surgical procedure to remove her from her sister's corpse. Still, she refused, saying, "As we came together, we shall also go together." Six hours later, she passed away [8]. A pair of Siamese twins born in India are shown in Figure 1.

Nomenclature

**Figure 2:**
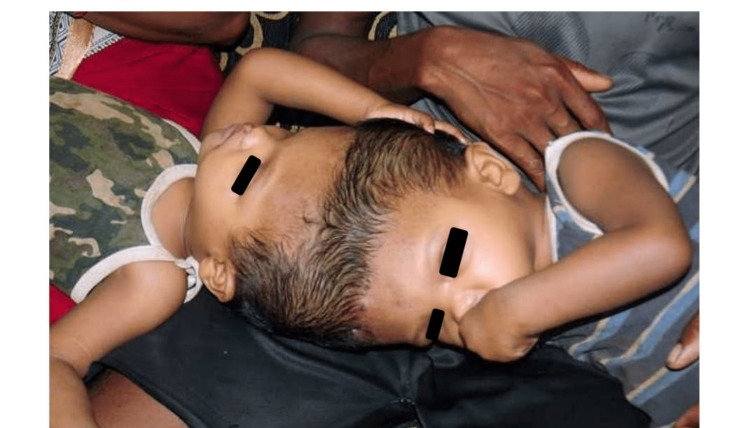
Craniopagus twins Image credit: Author Aabhas Bindlish

After day 13 of fertilisation, cleavage or axis duplication leads to conjoined twinning. They are classified based on the fusion site (Table 1) [9]. A pair of craniopagus twins are shown in Figure 2.

**Table 1:** Nomenclature of conjoined twins

TYPES	DESCRIPTION
Cephalopagus	The vertex and umbilicus are fused together. They have a single conjoined cranium that has either one composite face or two faces on the opposite side of the head. The upper digestive tract, heart, liver, and thorax are united together until the Meckel diverticulum, where they separate. Typically, there are four limbs and legs. Usually, these twins are not viable [10].
Thoracopagus	They are the most prevalent of all the kinds and are identical twins joined at the chest and umbilicus. However, they have a low survival rate [11].
Omphalopagus	Omphalopagus twins are conjoined twins that share a portion of the abdominal wall and gastrointestinal tract. If effectively separated, these kinds of twins have the best prospects of survival [12].
Ischiopagus	Ischiopagus twins have varying degrees of severity in the spine, central nervous system, gastrointestinal, and genitourinary tracts. It is a surgical difficulty to separate them [13].
Parapagus	Conjoined twins known as parapagus twins are fused ventrolaterally and are found side by side. Typically, the umbilicus, abdomen, and pelvis of this kind of conjoined twins are shared. There may be one symphysis pubis and one or two sacra in the conjoined pelvis. The genitourinary tract, gallbladder, liver, and pancreas are other organs that are frequently shared. The lower gastrointestinal tract (single colon and rectum) is also frequently shared [14].
Craniopagus	There are two types of craniopagus: partial and complete. In the partial version, the union has a limited scope, especially in terms of its depth, and it is assumed that both offsprings would survive and go on to lead everyday lives after separation. The two brains can be thought of as existing within a single cranium in the complete form, which has three identified types, and several gross intracranial abnormalities start to appear. These include a massive vascular anomaly, a deformation of the base of the skull, and a deformity and displacement of the cerebrum [15].
Pygopagus	They share the spinal cord, genitourinary system, gastrointestinal system, and sacrum to varying degrees and are linked in the sacral region. They represent a set of conjoined in which the embryonic axis' caudal separation was imperfect [16].
Rachipagus	Conjoined twins of the rachipagus variety are incredibly rare. They are separated from one another and linked in the dorsal aspect. The spinal cords may be shared as a result of the occiput fusing with various vertebral column segments. Above the sacrum, the fusion comes to an end. Legs and faces seem to be separate [17].

Medical advancements

Many conjoined twins can now be divided because of improvements in medical technology and care, usually following lengthy operations, provided that each twin has separate and healthy essential organs. Advances in medicine, imaging, and surgery have allowed for earlier, more precise representations of the anatomy, which has given a team of professionals more time to plan for their complex care. Patients with severe congenital heart disease have been simulated using prenatal imaging, diagnostic, and risk categorization algorithms that are used to create care plans which are intended to reduce risk and produce positive results. However, the literature on the delivery room and postnatal treatment of conjoined twins is scant and is only found in papers on omphalo-ischiopagus twins and embryology [18].

The fast expansion of simulation in health organizations has given us the chance to conduct difficult, uncommon, and dangerous medical and surgical techniques in a safe environment that is, on a simulator, not an actual patient. With the development of sophisticated simulation tools, surgeons and surgical teams are now able to perform SbCR's that are not only realistic but also replicate the precise anatomy of individual patients, improving patient care results [19].

The use of 3-D printing in medicine is not new. It has been utilized for many different things over the years, including splints, implants, and models for other procedures like heart surgery. Over the past ten years, 3D Systems has worked with various attorneys on dozens of conjoined twin cases [20].

However, it is highly difficult or impossible to decide on the separation of twins when they are united with a critical organ, such as the brain or heart, because doing so could result in death or brain damage. The likelihood and outlook for a separation surgery depend on the degree of organ sharing. Rarely, vascular shunts and cross-circulation between twins could result in a catastrophic outcome if they are not promptly diagnosed and treated. This syndrome is comparable to intrauterine twin-to-twin transfusion, in which one of the twins donates blood while the other receives it. Conjoined twins also occasionally share vital organs. Therefore, it may be possible to perform sacrifice surgery on one sibling to preserve the other. However, this choice has been the subject of significant medical, ethical, legal, religious, media, and public discussions [21].

Embryological discussion

The female reproductive system's anatomical structure and physiological functioning predispose to the conception of a single embryo, which develops into a fetus and is born naturally as a normal neonate at term after implantation. However, various disorders can sometimes disrupt this series of processes. Multiple pregnancies are one example of such a disorder, which is commonly and incorrectly referred to as twin pregnancies because there can be more than two fetuses. Ovulation disruptions can result in the release of multiple oocytes, which, when combined with sperm cells, form independent zygotes and, eventually, polyzygotic fetuses. Embryonic regulation is a characteristic of an embryo's early development. It can result in the action of genetically identical (monozygotic) twins. This is not considered a pathology, partly because subsequent embryonic development, labour, and postnatal development are frequently normal. However, significant developmental anomalies can occasionally occur when an embryo is damaged at an early stage of development, and subsequent attempts to repair it through biological regulation are not entirely normal. One such example is the formation of fetuses connected in various ways, known as conjoined or Siamese twins. Such a situation is always a severe pathology and leads to several issues, primarily medical in nature, regarding the prospects for such children's survival and their quality of life [22].

Ovarian biopsies from women between the ages of 18 and 52 revealed the presence of multi-ovular follicles, the vast majority of which (97.1%) were binovular, i.e., they contained two oocytes. The most likely cause of the development of binovular follicles is the failure of two distinct germ cells to be segregated by granulosa cells in the initial stages of folliculogenesis. The prior distance between the two germ cells may therefore influence the zonal fusion pattern [23]. Then, depending on how each oocyte develops and forms its zona, it will either produce conjoined gametes with a single, intact zona or two oocytes with distinct zones that are united in a specific area. In fact, a binovular follicle may include two oocytes that have not fused in the zonal zone and are surrounded by separate cumulus cells. According to researchers, two neighbouring oocyte-cumulus complexes that could be easily differentiated were found in nine follicular aspirates. On the other hand, two oocytes with distinct corona radiata were discovered inside a single cumulus complex in another instance [24,25]. According to research conducted previously, 61 out of 251 laparoscopies, that is, around 24% laparoscopies produced polyovular follicles, with five of those cases having three or more oocytes. The present analysis focuses on conjoined oocytes as the most trustworthy evidence for real binovularity. Still, it cannot be ruled out that some of these oocytes came from distinct follicles [26].

Evaluation

The best early-pregnancy diagnosis method is still first-trimester ultrasonography. Embryological abnormalities, tissue characterisation, and determining the kind of conjunction can all be aided by prenatal magnetic resonance imaging. Postnatal magnetic resonance imaging should be guided by prenatal imaging [27]. If necessary, surgical pre-planning and subsequent separation may benefit from modern techniques like 3D printing [28].

Statistics

Conjoined twins are rare worldwide; their prevalence is only estimated at 1.47 per 100,000 live births [29]. One of the rarest congenital developmental flaws in kids is this one. Additionally, it is hypothesized that the frequency may differ in various parts of the world or even among different ethnic groups. Conjoined twin statistics around the world vary depending on population sizes studied, whether conjoined twins are included or excluded in stillbirths, and whether or not women are willing to terminate pregnancies if such pathology is found. About 40-60% of conjoined twins who are born are stillborn [30]. Additionally, the score is unusually high in spontaneous miscarriages. In addition, Siamese twins have additional congenital flaws that are unrelated to the shared organs [31].

Anomalies associated with conjoint twins

It is essential to consider the mechanisms and diseases of monozygotic twin embryological development while seeking to explain how conjoined twins are generated [32]. The anomalies associated with conjoint twins are given in Table 2.

**Table 2:** Anomalies associated with conjoint twins

Thoracic anomalies	Dextrocardia {in thoracopagus and di-cephalic parapagus twins}, congenital diaphragmatic hernia, anomalous pulmonary venous drainage
Gastro-intestinal anomalies	Meckel’s diverticulum, bowel atresia, anomalous hepatic venous drainage [33]
Genito-urinary anomalies	Duplex system, renal dysplasia, pelvic-ureteric junction obstruction, vesico-ureteric junction obstruction [34]
Musculoskeletal anomalies	Congenital dislocation of the hip, clubfeet, Vertical tail Scoliosis

Surgical considerations

The type of surgery carried out depends on the location, degree of intricacy, and organ types shared in the conjunction. The surgical separation is customized for each patient and necessitates meticulous planning and a multidisciplinary approach. Regular, thorough interdisciplinary meetings are held by the surgeons during the preoperative evaluation period, during which imaging plays a crucial role in determining if the pediatric surgeons need support from cardiothoracic, orthopaedic, or urologic surgery experts. Intensive care and surgical theatre nurses, therapists, surgical theatre staff, management, and public relations staff are all invited to the sessions. During preoperative preparation, two distinct surgical teams are established, each in care of one of the twins; a skilled general paediatric surgeon is normally in charge of overall coordination. Planning the separation of the twins requires a precise evaluation of anatomy and blood supply. The anesthesiologist faces a great deal of difficulty while providing anaesthesia for conjoined twin surgery, whether it is for separation or before. The importance of treating each twin as a distinct individual must be emphasised [34]. For proper anaesthetic management and to enable early closure and separation of these vessels during surgery to prevent hypovolemic shock from blood volume loss through these shared veins, evaluation of vascular shunts and cross-circulation is crucial.

The multidisciplinary team reviews the data to plan separation once all organ systems have been assessed and vascular regions have been determined. Decisions are made on the distribution of the twins' organs, the order in which the organs are removed, anaesthesia, the monitoring of vital signs, wound closure, as well as preoperative tissue expansion, and postoperative care. To make sure that every member of the team is comfortable with their roles and the general plan of the surgery, the authors advise using diagrams, three-dimensional organ models, and thorough rehearsals of the separation procedure. This will help the actual operation go as smoothly as possible. Two different teams simultaneously anaesthetize the twins, and the surgeon in charge supervises as various speciality surgeons carry out their assigned surgical operations. The teams operate separately to perform reconstructive surgery, hemostasis, and wound closure once the twins have been physically separated. One group then transfers into an adjacent surgical theatre. During the operation, the twins must be cautiously watched since blood loss could hasten their decline and instability. There should be sufficient venous access, and pulse oximetry should be employed in conjunction with arterial pressure and continuous central venous monitoring, capnography, and regular blood gas analysis. Additionally, the electrocardiogram and urine output are carefully monitored. Following surgery, patients are kept under rigorous observation with prolonged ventilation, sedation, and monitoring for signs of sepsis, as well as fluid and electrolyte balance. Pediatric surgeons face a difficult task in separating conjoined twins. The radiologist faces a special challenge when interpreting preoperative imaging results. For surgical planning and prognosis, an imaging method that precisely characterizes anatomic fusion, vascular anomalies, and related abnormalities is essential. Accurate preoperative imaging facilitates conjoined twin separation [28].

Following the first diagnosis, conjoined twins are usually delivered via cesarean section. Siamese twins frequently have developmental problems with their neurological, urogenital, circulatory, and musculoskeletal systems. Many Siamese twins pass away in the neonatal stage, including those who pass away during a separation procedure. The type of connection and shared organs, as well as the timing and accuracy of surgery and nonsurgical therapy, are the significant factors determining their life. Conjoined twins frequently die after surgery due to postoperative stress, associated deformities, and a shortage of material to hide body flaws or infections. Sharing vital organs makes separation difficult. A planned procedure has a higher chance of being successful than a life-saving one. This is primarily due to the fact that all investigations are completed before a scheduled procedure in order to determine the exact course of the connection between twins, which reduces the likelihood of discovering unexpected anatomical features during the surgery. The clinical analysis of children treated in an institution in Krakow, Poland, from 1977 to 2006 highlights the challenges of separating Siamese twins. Nine twins perished, and ten were surgically separated from 19 sets of conjoined twins. After the surgery, one twin from each pair of eight twins survived, both twins from one team passed away, and both twins from one pair (split abroad) lived [34].

## Conclusions

In the end, we can say that conjoined twins/Siamese twins are embryological abnormality that occurs in fetuses in the mother’s womb. Due to more research being conducted on this matter, a higher amount of anomalies are being discovered. As these anomalies are being studied, more people are getting to know about them in detail. Also, new technologies and medicines have been in production and are designed to help their chances of birth. Recent technologies have made it possible for them to stay alive for a longer time and live a prosperous life. The ever-evolving surgical interventions are making it better to provide them and their mothers with better quality of living, with surgical time reducing over the years and the medicinal improvements that are helping the mother to deliver normally. The new technologies such as 3-D printing and Artificial intelligence are of utmost help as they provide the surgeons the chances to make mistake on a demo model rather than make mistakes on an actual patient in real time. It must be noted that planning and performing these surgeries require precise knowledge and evaluation before the beginning of the separation surgeries. The ratio of birth to death is increasing each year.
